# Self-assembled organic–inorganic magnetic hybrid adsorbent ferrite based on cyclodextrin nanoparticles

**DOI:** 10.3762/bjoc.8.215

**Published:** 2012-11-01

**Authors:** Ângelo M L Denadai, Frederico B De Sousa, Joel J Passos, Fernando C Guatimosim, Kirla D Barbosa, Ana E Burgos, Fernando Castro de Oliveira, Jeann C da Silva, Bernardo R A Neves, Nelcy D S Mohallem, Rubén D Sinisterra

**Affiliations:** 1 Centro Federal de Educação Tecnológica (CEFET-MG), Campus VII, Timóteo, MG, Brazil 35183-006; 2 Universidade Federal de Juiz de Fora (UFJF), Governador Valadares, 35010-177, MG, Brazil; 3 Laboratório de Encapsulamento Molecular e Biomateriais (LEMB) – Departamento de Química, Instituto de Ciências Exatas, Universidade Federal de Minas Gerais (UFMG), Belo Horizonte, 31270-901, MG, Brazil; 4 Universidad Nacional de Colombia Bogotá – DC, Colombia; 5 Departamento de Física, ICEx, Universidade Federal de Minas Gerais (UFMG) Belo Horizonte – MG, 31270-901, Brazil; 6 Laboratório de Materiais Nanoestruturados, Departamento de Química, ICEx, Universidade Federal de Minas Gerais (UFMG) Belo Horizonte – MG, 31270-901, Brazil

**Keywords:** assembled particles, colloids, cyclodextrin, ferrite, hybrid materials

## Abstract

Organic–inorganic magnetic hybrid materials (MHMs) combine a nonmagnetic and a magnetic component by means of electrostatic interactions or covalent bonds, and notable features can be achieved. Herein, we describe an application of a self-assembled material based on ferrite associated with β-cyclodextrin (Fe-Ni/Zn/βCD) at the nanoscale level. This MHM and pure ferrite (Fe-Ni/Zn) were used as an adsorbent system for Cr^3+^ and Cr_2_O_7_^2−^ ions in aqueous solutions. Prior to the adsorption studies, both ferrites were characterized in order to determine the particle size distribution, morphology and available binding sites on the surface of the materials. Microscopy analysis demonstrated that both ferrites present two different size domains, at the micro- and nanoscale level, with the latter being able to self-assemble into larger particles. Fe-Ni/Zn/βCD presented smaller particles and a more homogeneous particle size distribution. Higher porosity for this MHM compared to Fe-Ni/Zn was observed by Brunauer–Emmett–Teller isotherms and positron-annihilation-lifetime spectroscopy. Based on the pKa values, potentiometric titrations demonstrated the presence of βCD in the inorganic matrix, indicating that the lamellar structures verified by transmission electronic microscopy can be associated with βCD assembled structures. Colloidal stability was inferred as a function of time at different pH values, indicating the sedimentation rate as a function of pH. Zeta potential measurements identified an amphoteric behavior for the Fe-Ni/Zn/βCD, suggesting its better capability to remove ions (cations and anions) from aqueous solutions compared to that of Fe-Ni/Zn.

## Introduction

Organic–inorganic hybrid materials (HMs) are often prepared by assembling organic and inorganic molecules based on electrostatic interactions or chemical bonding between them, which will leads to an unpredictable stoichiometry [1]. The structures and properties of HMs depend on the nature of both components, organic and inorganic, and also on the synthesis process, which can be carried out by metal or organic hydrolyses [2–3]. Controlling the method of HM synthesis could lead to a predictable crystal structure and homogeneous particle size distribution [4]. Additionally, surface properties could be modulated by selecting an appropriate organic molecule with desirable functional groups in its structure. In particular, magnetic organic–inorganic hybrid materials (MHMs) have attracted considerable attention based on their multifunctional and biocompatible properties [2,5–6]. Moreover, MHMs can present a greater number of applications than nonmagnetic hybrid materials when their suspensions are used.

These MHMs systems are susceptible to external magnetic fields because of the strong magnetic interactions existing among the magnetized particles, which are able to modify the colloidal and rheological properties of the solutions. Thus, functions based on their magnetic characteristics such as conduction and accumulation in a system under an external magnetic field can be realized. In this sense, colloidal systems of MHMs have shown an increasing number of applications in many different technologies, including ferroﬂuids [7], magnetic separators [8], magnetic resonance imaging [9], hyperthermia [10], and water treatment [4].

Nickel-zinc ferrites (Fe-Ni/Zn) are one of these versatile magnetic materials, since these systems present high magnetic saturation, Curie temperature and chemical stability. Additionally, low coercivity and biodegradability have been observed in Fe-Ni/Zn, being an interesting part of the inorganic component in the MHM structure [11]. The organic molecule in the MHM should present specific characteristics in order to improve the material application, including available binding sites, to bond or interact through intermolecular forces with the inorganic matrix, and high surface area, which is important to improve the MHM adsorption and adhesion properties. In order to design a MHM based on Fe-Ni/Zn with adsorption properties for environmental use, cyclodextrins (CDs) can be associated with the inorganic matrix as an interesting strategy, since these macromolecules have been used for several devices with different properties, from light-responsive matrices to molecular recognition materials [12–13].

CDs are oligosaccharides commonly formed by six, seven or eight α(1→4) linked-D-glucopyranoside units, named αCD, βCD and γCD, respectively. These macromolecules have a rigid and well-defined structure with a toroidal shape, in which a variety of organic and inorganic guest molecules can be inserted into their cavities, resulting in the formation of inclusion complexes [14–17]. Beyond these characteristics, it has been reported in the literature that CDs self-assemble into large aggregates [18–20], suggesting their uses as size-modulator molecules [21], similar to other amphiphilic molecules [22–25]. Based on these interesting properties for both systems, CDs and Fe-Ni/Zn can be used to synthesize a MHM with large number of applications. Moreover, due to the arrangement of CDs, their primary and secondary hydroxy groups may be able to interact with a ferrite structure, through covalent bonds or also by intramolecular interactions. These hydroxy groups could improve the adsorption properties of the MHM by including guest molecules in CD cavities or also by allowing the assembly process. Recently, adsorption materials have become one of the most versatile and widely used technologies to remove heavy metals from industrial wastewater, including chrome in its different oxidation states [4,26]. Although activated carbon has been frequently applied for this purpose, magnetic adsorbent materials have also demonstrated their potential applicability in the past few years, because of their easy separation properties.

Herein, Fe-Ni/Zn and the MHM prepared by using Fe-Ni/Zn and βCD (Fe-Ni/Zn/βCD) were synthesized by adapting a method previously described in the literature [7]. These magnetic materials were characterized in the solid state by X-ray powder diffraction (XRD), Fourier transform infrared spectroscopy (FTIR), and thermal analysis (TG/DTA), and by their magnetic behavior in aqueous suspension (see Supporting Information File 1). Fe-Ni/Zn and Fe-Ni/Zn/βCD nanoparticles morphologies were investigated in the solid state by scanning electronic microscopy (SEM), transmission electronic microscopy (TEM), and atomic force microscopy (AFM). Size distribution and colloidal suspension stability were characterized by dynamic light scattering (DLS) and sedimentation kinetic studies by using UV–vis spectroscopy. Ferrite binding sites were characterized by zeta potential (ZP) and potentiometric titration. The free volume for both materials was evaluated by Brunauer–Emmett–Teller isotherm (BET) and positron-annihilation-lifetime spectroscopy (PALS). Thus, considering the advanced functional properties achieved by βCD insertion in the inorganic matrix identified by the above experiments and the MHM capability to self-assemble, the adsorption properties for both ferrites using chrome ions (Cr^3+^ and Cr_2_O_7_^2−^) in aqueous solutions were tested and evaluated by ZP measurements.

## Results and Discussion

### Fe-Ni/Zn and Fe-Ni/Zn/βCD size characterization

**Figure 1:**
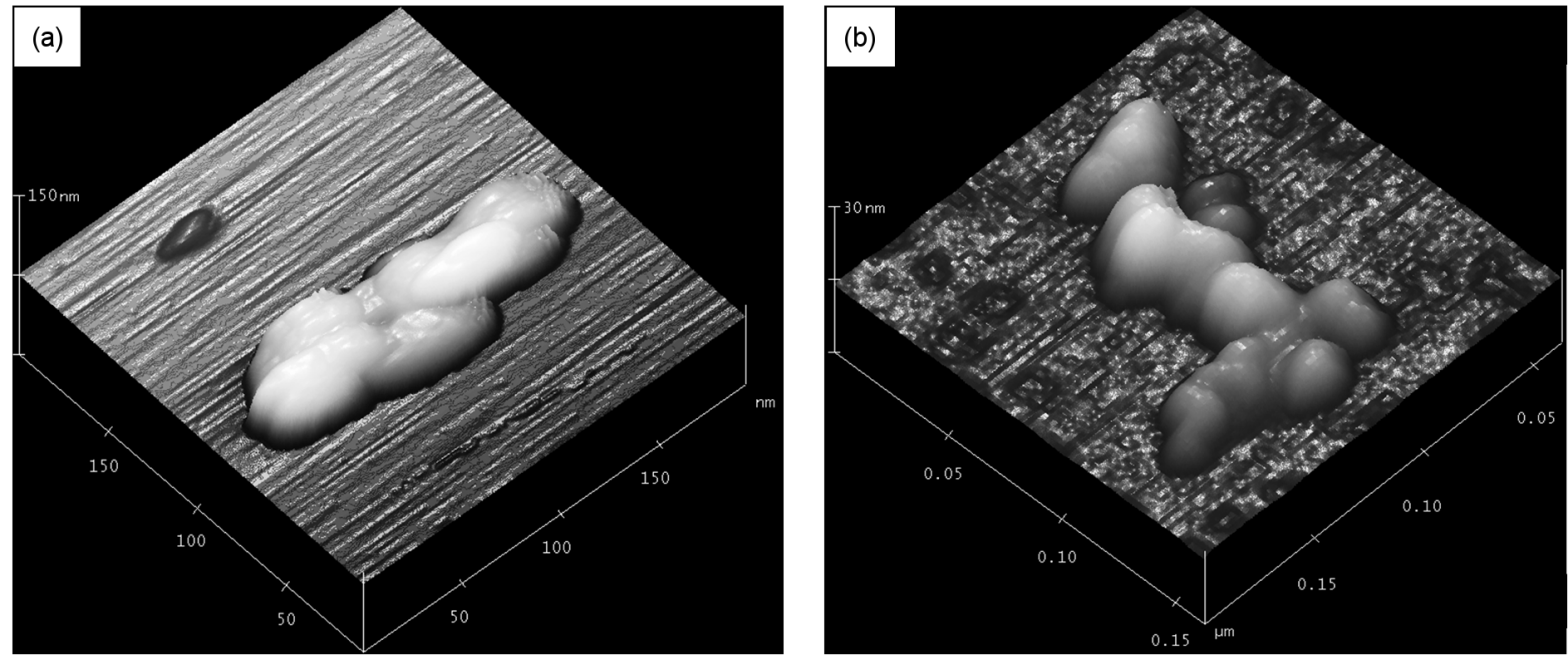
AFM images of (a) Fe-Ni/Zn and (b) Fe-Ni/Zn/βCD.

In order to investigate the Fe-Ni/Zn and Fe-Ni/Zn/βCD morphology and size distribution at different aggregation levels, these materials were investigated in the solid state by SEM, TEM and AFM and also in aqueous suspension by using DLS. AFM images of Fe-Ni/Zn and Fe-Ni/Zn/βCD are presented in Figure 1. Based on these images, nanoparticles below 100 nm were verified for these materials. Looking closer, Fe-Ni/Zn/βCD presents smaller nanoparticles in a range of 20 to 50 nm, while the Fe-Ni/Zn consisted of domains higher than 50 nm. The smaller particles observed for the Fe-Ni/Zn/βCD particles could be due to the size-modulator effect of CDs [19–20,27–28], which could act as a nanoreactor for ferrite synthesis, preventing the particles growing during the ferrite synthesis (nucleation process).

**Figure 2:**
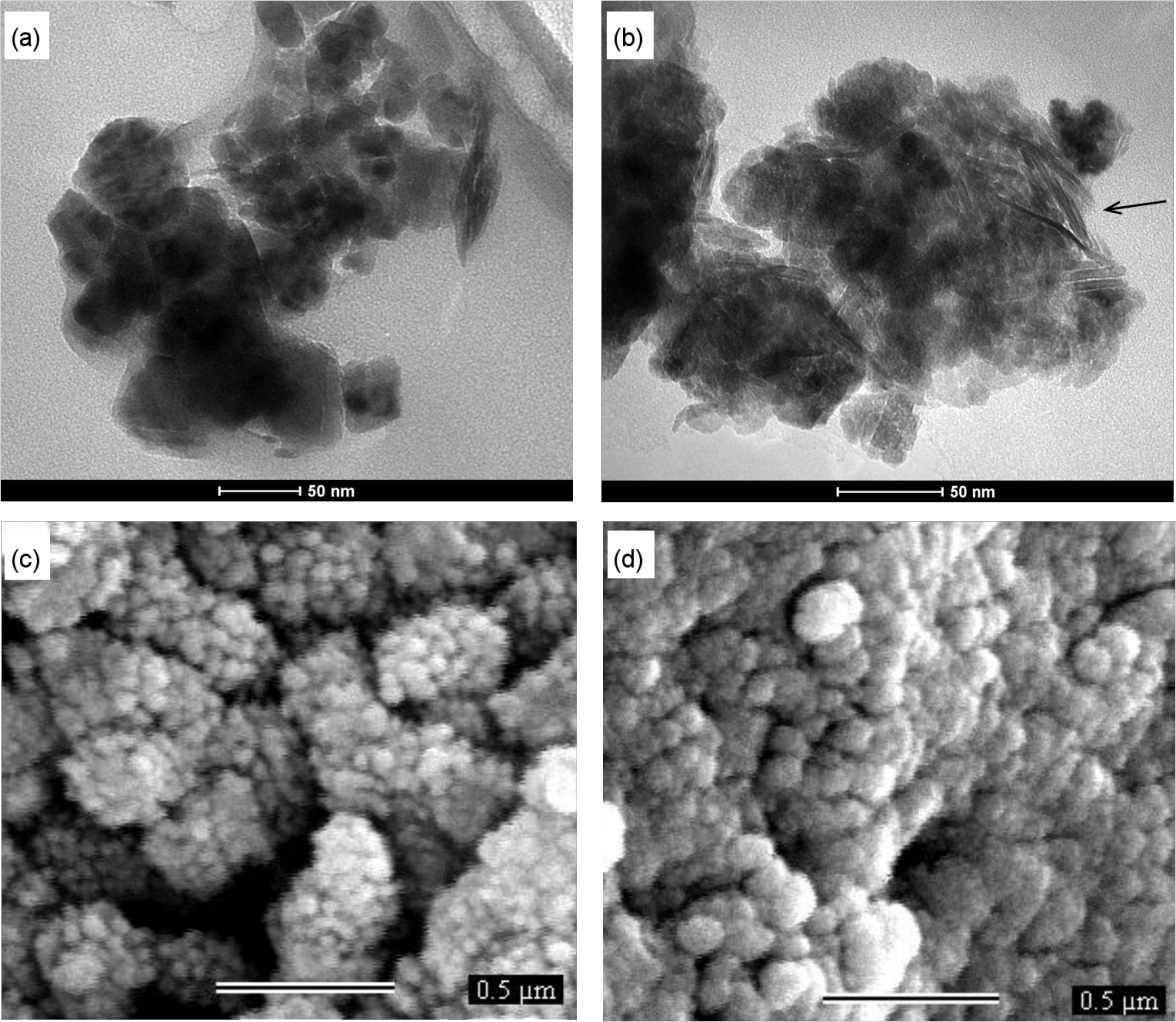
TEM images of (a) Fe-Ni/Zn and (b) Fe-Ni/Zn/βCD and SEM images of (c) Fe-Ni/Zn and (d) Fe-Ni/Zn/βCD.

In order to gain insight into the microstructure of these assembled materials, SEM and TEM were also carried out. TEM images of the magnetic materials, demonstrating their structure from the nanoscale level up to the aggregate particles, are shown in Figure 2a and Figure 2b. In addition, the TEM image for the Fe-Ni/Zn/βCD showed aligned structures in the nanoparticle matrix, which can correspond to the assembled βCD structures in the MHM. These lamellar self-assembled structures are similar to the βCD crystals described previously in the literature [29], confirming the presence of the macromolecule in the ferrite nanoparticles matrix. SEM images, Figure 2c and Figure 2d, demonstrate larger assembled particles (above 500 nm). It is interesting to note that when βCD is used to prepare the ferrite nanoparticles, size domains are smaller than those observed for the material prepared without the macromolecule. These results corroborate the hypothesis considering the size-modulator effect of βCD proposed previously based on the analysis of the AFM images.

**Figure 3:**
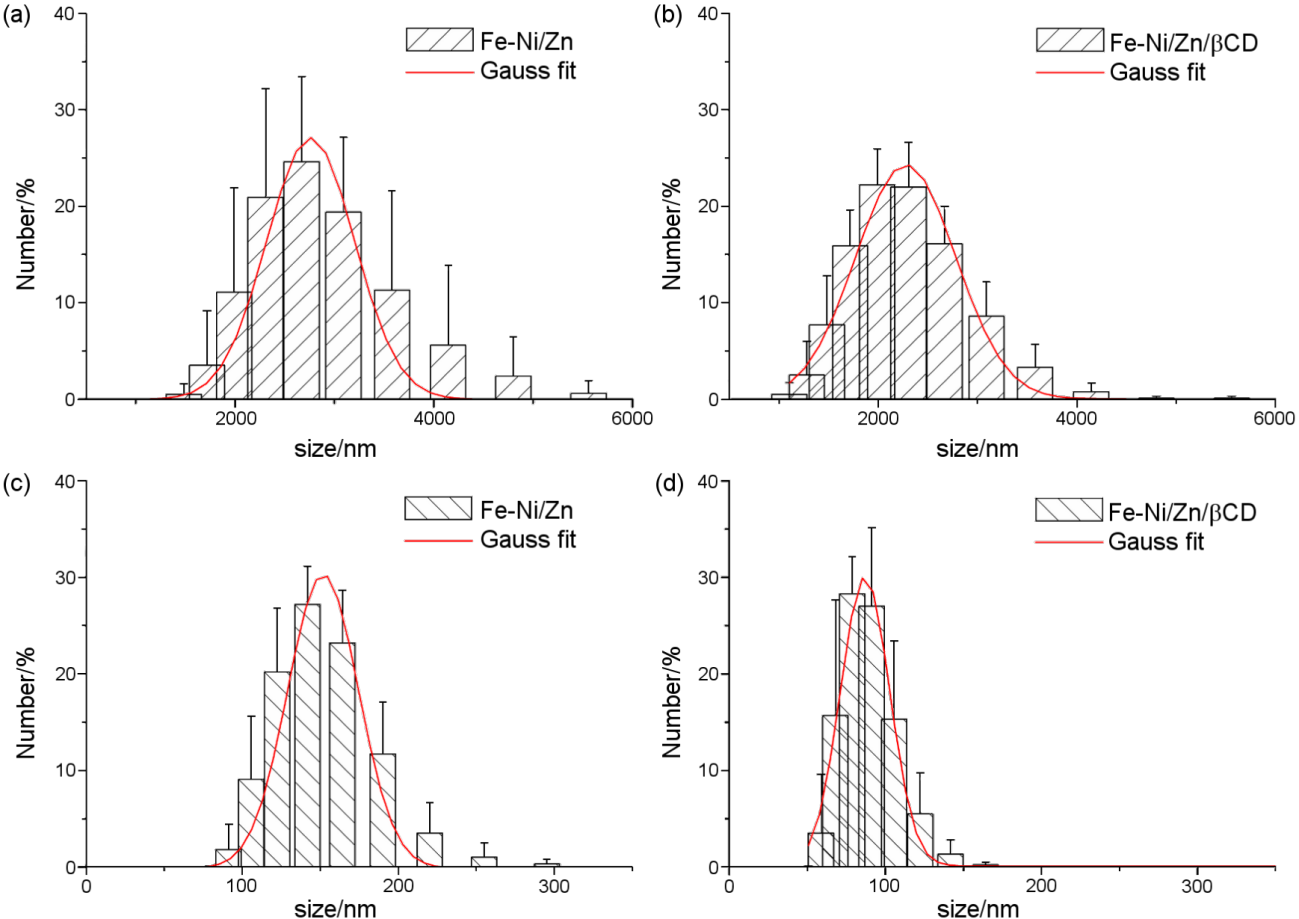
DLS measurements for before (a) and (b) and after (c) and (d) the sonication process.

Fe-Ni/Zn and Fe-Ni/Zn/βCD were also analyzed in aqueous suspensions, at pH 7, (Figure 3). The ferrite aggregation process was confirmed, Figure 3a and Figure 3b, in which particle size distributions of about 2.8 and 2.3 μm were observed for Fe-Ni/Zn and Fe-Ni/Zn/βCD, respectively. In the presence of βCD a more homogeneous size distribution was verified, suggesting that βCD is able to minimize the ferrite coalescence process in aqueous solution. The ferrite aggregation process may occur in low zeta potential values (−10 mV, see zeta potential data) and is insufficient to avoid van der Waals attraction [30]; however, at pH 7 this self-assembly behavior can also be verified. Evidence for the ferrite aggregation process is observed in aqueous suspensions when these materials are sonicated, Figures 3c and Figure 3d, in which the assembled structures are disrupted in order to obtain the nanoparticles. Once again, Fe-Ni/Zn/βCD presented a smaller particles distribution (average size of about 85 nm) with a more homogenous size distribution than that prepared without βCD (Fe-Ni/Zn about 150 nm). These results are in accordance with those verified by AFM and TEM, in which Fe-Ni/Zn/βCD presented smaller nanoparticles than pure ferrite in the solid state. Although some discrepancy between the DLS and AFM particle size distributions has been observed, it has been previously reported in the literature for other systems [31–32].

### Textural characterization by gas adsorption

BET isotherms were carried out to investigate the free volume of the ferrites. Fe-Ni/Zn was able to adsorb gas at 115 cm^3^ g^−1^, showing a type IV isotherm by (Brunauer, Deming, Deming, and Teller) BDDT classification [33], which is characteristic of mesoporous materials, and its hysteresis loop closed at *p*/*p*_0_ = 0.2. Adsorbing capacity for the Fe-Ni/Zn/βCD was 125 cm^3^ of gas per gram, presenting an intermediary isotherm characteristic that changes from meso- to microporosity, and its hysteresis loop closed at *p*/*p*_0_ = 0.4, demonstrating that the presence of βCD implies a minor difficulty in adsorption.

Textural characteristics for the ferrite (Fe-Ni/Zn), which has a specific surface area of 127 m^2^ g^−1^, changed substantially with the inclusion of βCD in the inorganic matrix, increasing the specific surface area to 191 m^2^ g^−1^ (an increase of 50.4%). This surface area variation is mainly due to the microporosity increasing by the inclusion of the βCD in the inorganic matrix. Fe-Ni/Zn presented a fractal dimension (D) of 2.772, which increased to 2.934 with the macromolecule insertion, showing a significant growth in the surface roughness, since the D value of a surface with maximum roughness is 3. This result is in accordance with microscopy analysis in the solid state, which shows different morphologies for these two samples.

### Determination of free volume by PALS

The free volume was measured by positron probe through the PALS technique, where the longest component of the positron-lifetime spectrum, τ_3_ (the o-Ps pick-off lifetime), can be correlated with free volume holes in condensed matter. According to this model, τ_3_ grows leading to an increasing in the free volume radius, based on a spherical-cavity model [34–35]. The calculated free volume dimension has values of *V*_f_ = 69.9 Å^3^ and *V*_f_ = 115.9 Å^3^ for Fe-Ni/Zn and Fe-Ni/Zn/βCD, respectively; corresponding to an increase of 65% of the free volume (Δ*V*_f_ = 46 Å^3^). These results corroborate BET isotherm studies, which pointed out a greater porosity in the material prepared by using βCD.

#### Potentiometric titrations

**Figure 4:**
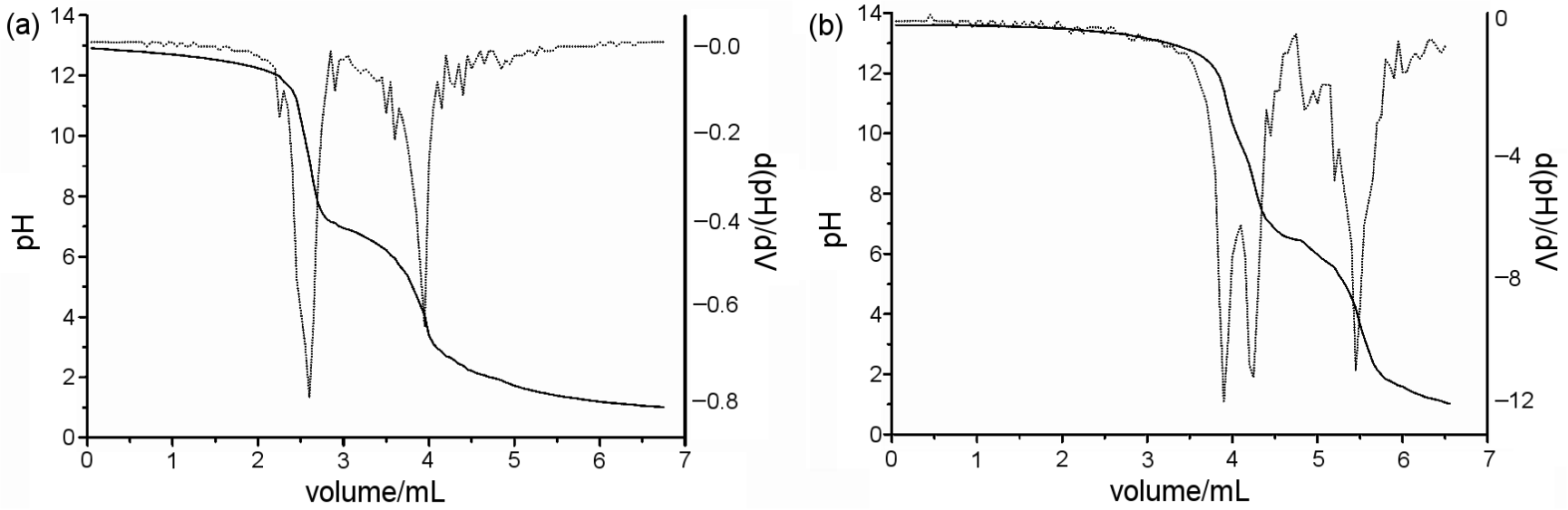
Potentiometric curves of the (a) Fe-Ni/Zn and (b) Fe-Ni/Zn/βCD.

Potentiometric titrations were recorded in order to determine the number of ionizable sites for the magnetic materials through the first derivative of the titration curve. As can be seen in Figure 4a and Figure 4b, the first derivative of the titration curves pointed out several transitions, demonstrating that ferrite could be considered a polyprotic acid, probably due to the ionization of the different groups on the ferrite surfaces. It can also be observed that the potentiometric curve for Fe-Ni/Zn/βCD exhibits at least four transitions, two in addition to the pure Fe-Ni/Zn. The pKa values of the ferrites were calculated by using the Henderson–Hasselbalch Equation 1, at the point where [A^−^] = [HA] [36]:

[1]
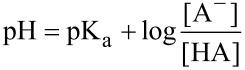


Transitions at pKa 6.5 and 13.5 were attributed to the ionization of ferrite groups in the MHM system, since Fe-Ni/Zn also presented close transition values (pKa 6.5 and 12.6). Thus, transitions at pKa 5.5 and 9.4 in the Fe-Ni/Zn/βCD were attributed to the ionization of βCD primary and secondary hydroxy groups. These results emphasize that βCD is part of the inorganic matrix, corroborating the data obtained by TEM, in which lamellar assembled structures were observed in the MHM matrix.

#### Zeta potential

**Figure 5:**
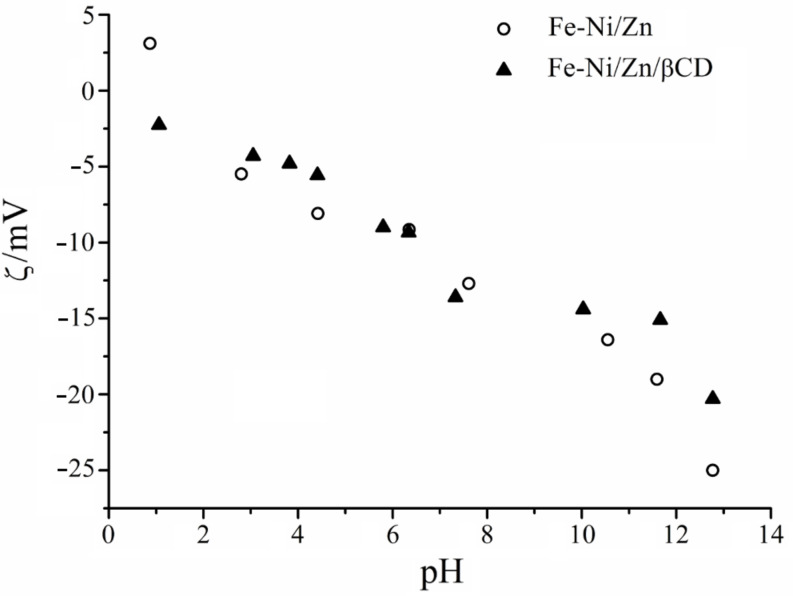
Zeta potential curves as a function of pH for the (○) Fe-Ni/Zn and (▲) Fe-Ni/Zn/βCD.

Particles in contact with an aqueous electrolyte solution acquire a surface charge as a result of adsorption or ionization processes. In order to evaluate the electrostatic repulsion between dispersed ferrite particles in solution, the electrical surface potential was evaluated by using ZP, computed from electrophoretic mobility measurements [37]. Figure 5 depicts the ZP variation as a function of the pH for the aqueous suspension of the ferrites, in which the solutions were tuned by using nitric acid or sodium hydroxide. These curves demonstrated that the zeta potential values are negative under the studied experimental conditions, and a pH dependency was also verified, once the ZP values become more negative increasing the pH of the aqueous suspensions.

The negative values in the pH range could be attributed to (i) ionization of R–OH groups after pH 6.5, which causes the formation of negative charges on the ferrite surface [38], and/or (ii) preferential adsorption of nitrate anions on the particle surface before this pH, since ferrites can become less hydrophilic upon protonation, and it is known that NO_3_^−^ exhibits a preferential adsorption on weakly hydrated surfaces [39]. The isoelectric point (IP) of ferrites is close to pH 7 [40], thus inflexions observed close to this value for both systems may be an apparent IP, which was masked by competition between the ionization of Fe–OH groups and preferential NO_3_^−^ adsorption. In other materials, negative ZP values have been also found in the pH range scanned from 1 to 13 [41–43].

Although the potentiometric titration demonstrated that ferrites present different transitions due to the different ionization sites, ZP titrations did not show the same behavior. A reasonable explanation for this phenomenon is based on the sensitivity of these analytical techniques. Potentiometry measures the electrical potential difference established through the electrode membrane, being directly dependent on the activity of H_3_O^+^ ions in the bulk solution, since its rate of diffusion is proportional to the concentration. ZP measurements obtained by Doppler electrophoresis are dependent on the Brownian diffusion of the particles in an electric field, which depends on the surface charge and size, and the size and charge polydispersities, as well as the aggregation state, among other phenomena; which are hard to control in a suspension material.

#### Sedimentation studies

**Figure 6:**
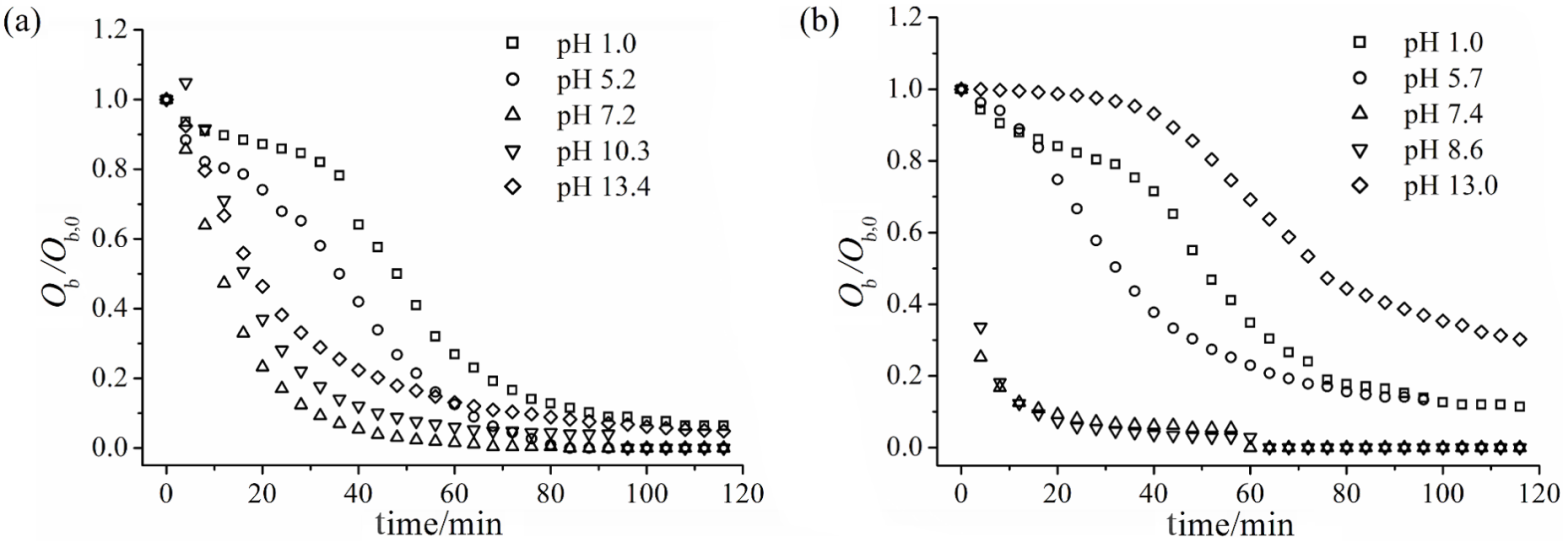
Relative optical obscuration (*O*_b_/*O*_b,0_) as a function of time for (a) Fe-Ni/Zn and (b) Fe-Ni/Zn/βCD.

Information about the colloidal stability of these ferrite suspensions can be inferred from the optical obscuration changes of the optic path by visible light at 700 nm as a function of time [40]. Figure 6 shows the obscuration (*O*_b_) relative to its absorbance at time *t* = 0 s (*O*_b,0_), plotted as a function of time for different pH values. Although the overall tendency of *O*_b_/*O*_b,0_ is to decrease over time due to particle sedimentation, the results obtained at different pH do not overlap, indicating that there is a clear pH dependence on the sedimentation rate. This pH dependence corresponds to the charge variation on the ferrite surface, which was also observed in the ZP titrations.

**Figure 7:**
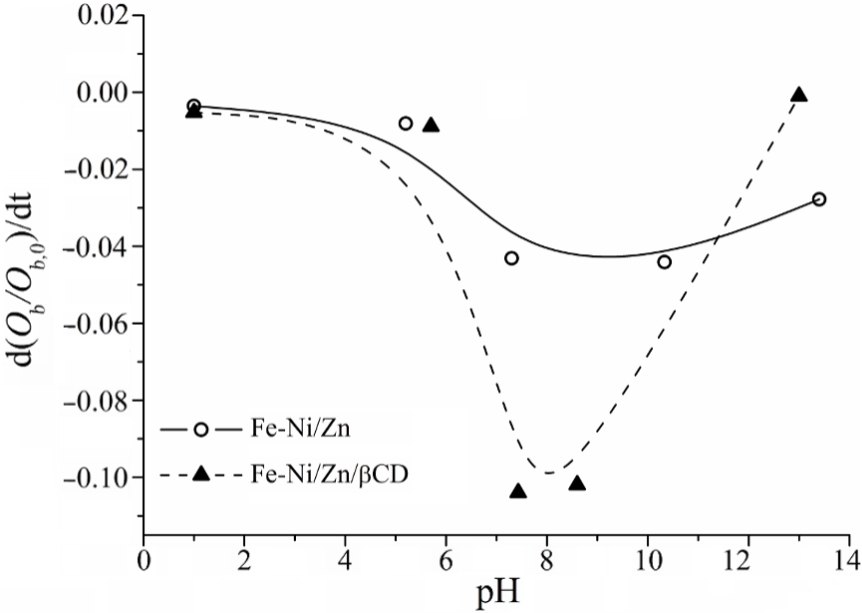
Obscuration curves for the Fe-Ni/Zn (○) and Fe-Ni/Zn/βCD (▲) as a function of pH.

Since light scattering and absorption properties upon sedimentation are complicated phenomena, only the initial slopes, *S* = d*(O*_b_/*O*_b,0_*)_t_→*_0_*/*d*t*, of the obscuration curves were used in order to evaluate the sedimentation rate, as suggested by Plaza et al. [40]. Figure 7 shows the initial sedimentation rate for both magnetic systems. It can be observed that *S* values are greater in magnitude for the Fe-Ni/Zn/βCD than for the Fe-Ni/Zn: once at neutral pH the initial sedimentation is faster. The maximum absolute *S* (i.e., |*S*|) can be related to the absence of electrostatic repulsion between the particles in the pH vicinity of the apparent IP, and particle aggregation could occur under these conditions. This is a direct consequence of the greater number of hydroxy groups in the Fe-Ni/Zn/βCD system.

At pH ≈ 1, both ferrites have approximately the same and slow rate of sedimentation, probably due to NO_3_^–^ adsorption. However, at pH ≈ 13, |*S*| is smaller for the Fe-Ni/Zn/βCD than for pure ferrite; in spite of the fact that the ZP results demonstrated the same values for both materials. The smaller particles and the greater presence of hydroxy groups of the Fe-Ni/Zn/βCD could be responsible for the slower sedimentation rate at this pH value.

#### Adsorption studies

**Figure 8:**
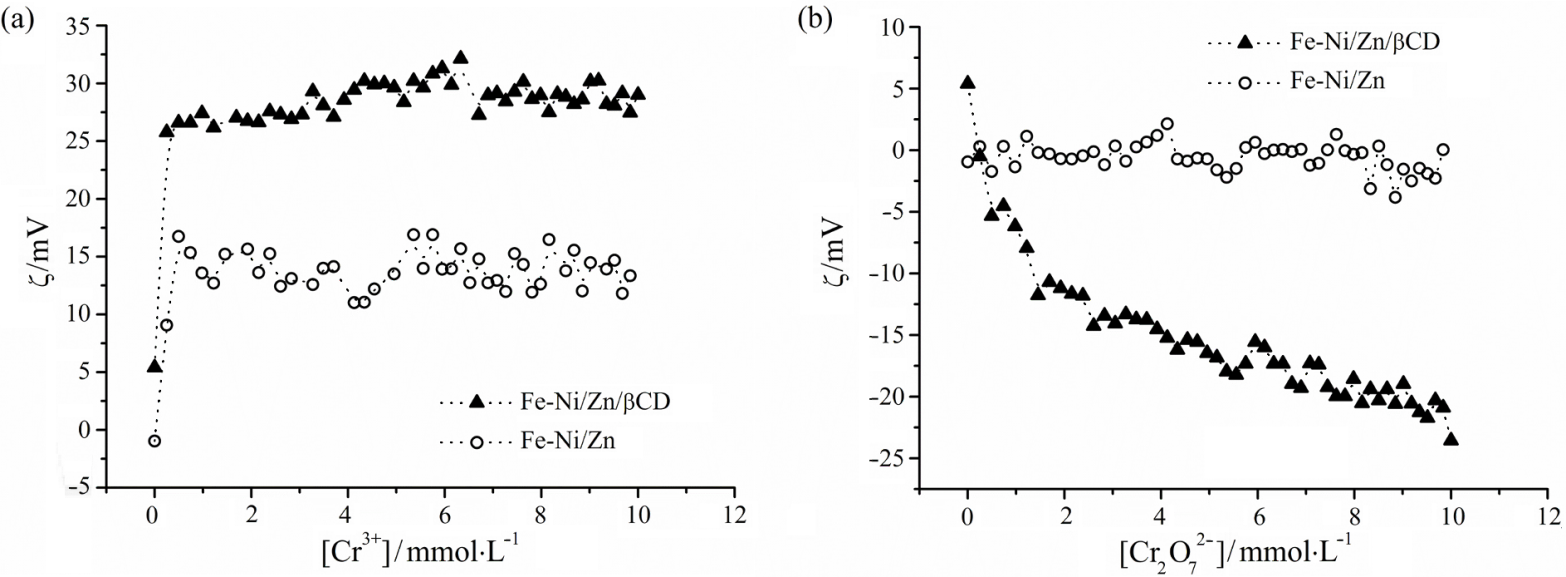
Adsorption curves of (a) Cr^3+^ and (b) Cr_2_O_7_^2−^ ions using Fe-Ni/Zn and Fe-Ni/Zn/βCD aqueous suspensions.

Adsorption studies were carried out in order to compare the ion adsorption capacity of these magnetic materials in aqueous solution. ZP measurements were used to gain insights into the adsorption processes of Cr^3+^ and Cr_2_O_7_^2−^ ions. Figure 8 shows how the ZP values change as a function of the ion concentration. The ZP titration curves indicated that the MHM is able to adsorb both ions (Cr^3+^ and Cr_2_O_7_^2−^) on its surface, since the ZP values (|ZP|) increase in their presence. These data suggest an amphoteric characteristic of Fe-Ni/Zn/βCD, which can interact with positive and negative species through ion–dipole or ion–ion interactions. Changes in the ZP values have been observed in the literature for different ferrites after the ion-adsorption process, indicating the affinity between them [44].

Comparing qualitatively the interaction involving the MHM with both ions, it is possible to observe its greater affinity for the Cr_2_O_7_^2−^ than the Cr^3+^, since for the latter a constant ZP value was observed above 0.4 mmol L^−1^, while a plateau was not reached for the Cr_2_O_7_^2−^ titration. This result also demonstrated that the MHM are able to adsorb more than 10 mmol of this anion per liter (maximum concentration tested), which is greater than that of approximately 0.4 mmol L^−1^ for the Cr^3+^ cation. The MHM capacity to adsorb Cr^3+^ ions (about 19 mg g^−1^ of MHM) was higher than that observed for MnFe_2_O_4_ ferrite (7.6 mg g^−1^ of MnFe_2_O_4_) to remove Cr^6+^ from aqueous solutions, described previously [4]. Thus, the inclusion of βCD in the inorganic matrix increased the ion adsorption, demonstrating the importance of choosing the organic component in a hybrid material carefully. Fe-Ni/Zn adsorption capacity for the Cr^3+^ cation was similar to that observed when βCD was used in the MHM. However without βCD, Fe-Ni/Zn was not able to adsorb the Cr_2_O_7_^2−^, since no change in the ZP values was verified, indicating that Fe-Ni/Zn and the anion did not interact in aqueous suspension.

## Conclusion

Hybrid magnetic nanoparticles based on ferrite and βCD were prepared and characterized in the solid state and in aqueous suspension, and their properties were compared with pure ferrite. Structural analysis pointed out that the synthesis approach was able to incorporate βCD in the ferrite matrix, which allowed us to keep the important βCD characteristics in the final magnetic material. Ferrite morphology in the solid state was affected by βCD insertion, probably due to its size and key role as modulator during inorganic nucleation. Ferrites presented at least three different organization scales, from the micrometric to the nanometric level, and on the nanometric scale it was possible to verify the material organization and particle size distribution. Lower domains, and greater specific area and free volume were observed for the Fe-Ni/Zn/βCD material. On the micrometric level, both ferrites have comparable behavior, with quite similar size distribution and ZP values, although Fe-Ni/Zn/βCD presents a slower sedimentation at high pH values. Finally, the MHM is more versatile for the adsorption of ions in aqueous solution than Fe-Ni/Zn due to its pronounced amphoteric characteristic, which was obtained by βCD incorporation in the inorganic matrix.

## Experimental

### Materials and Methods

#### Reagents and ferrite synthesis

βCD was obtained from Xiamen Mchem, Xiamen (China). Salts used for the ferrite synthesis (FeSO_4_·7H_2_O, NiSO_4_·6H_2_O and ZnSO_4_·7H_2_O) were obtained from Merck Laboratory and used without further purification. Magnetic nanoparticles of nickel/zinc (Fe-Ni/Zn) and nickel/zinc/cyclodextrin (Fe-Ni/Zn/βCD) were prepared by coprecipitation reaction of their metal sulfates at 80 °C and pH > 12 (with a sodium hydroxide concentration of 15 g L^–1^), following the method described in the literature [21]. In the Fe-Ni/Zn/βCD synthesis, 5.0 g L^–1^ of βCD was used during the preparation process. Solid magnetic materials obtained were washed with hot distilled water and filtered, and then the freeze-dried material was used in the solid-state characterization. Part of the ferrite was kept in water to investigate the properties of the aqueous suspension.

#### Microscopy analysis

Scanning electron microscopy (SEM) was performed in a JEOL, JSM 840A at 4–10 KV in which samples were covered with a thin gold layer, for electronic contrast. Transmission electron microscopy (TEM) images were obtained in a FEI TECNAI G2 with a thermo-ionic gun at 200 kV. Atomic force microscopy (AFM) images were obtained with a Nanoscope IV MultiMode from Veeco Instruments operating in intermittent contact (tapping) mode, with standard Si probes. Phase-contrast images were acquired simultaneously with topographic images by monitoring, with a lock-in amplifier, the phase lag between the oscillation driver and the actual response of the cantilever.

#### Dynamic light scattering

Fe-Ni/Zn and Fe-Ni/Zn/βCD average hydrodynamic diameter was measured in a Malvern Zetasizer Nano Series ZS particle analyzer, by using polyethylene square cells. All suspensions were prepared by using Milli-Q water before and after the sonication process by using a Sonics Vibra Cell coupled with a microprobe, at 25% amplitude for 5 min. Samples were measured by monochromatic light (10 mW He–Ne laser, wavelength 632.4 nm) and the scattered light intensity was measured in an angle of 173°. Hydrodynamic diameters were measured five times independently and each one was obtained as the mean of 30 counts.

#### Gas adsorption/desorption isotherm studies

Textural characteristics of Fe-Ni/Zn or Fe-Ni/Zn/βCD samples were determined through nitrogen gas adsorption (Autosorb–Quantachrome Nova 1200) at liquid-nitrogen temperature. Nitrogen gas was used with a 25-point adsorption–desorption cycle. Samples were outgassed at 100 °C for 3 h before each analysis, and experiments were carried out in triplicate. The specific surface area and fractal dimension were obtained by the application of the Brunauer–Emmett–Teller (BET) equation and the Neimark–Kiselev (NK) methods.

#### Positron-annihilation-lifetime spectroscopy

PALS measurements of Fe-Ni/Zn or Fe-Ni/Zn/βCD were performed at 294 K by using a conventional fast–fast coincidence system (Ortec) with resolution time of 260 ps given by the ^60^Co prompt curve. The ^22^Na positron source, with approximately 20.0 µCi activity, was sandwiched between two 7.6 μm thick kapton foils, and the source correction was approximately 10%. The lifetime spectra (minimum of three measurements per sample) were satisfactorily resolved into three components by the Positronfit-Extended program [45], leading to intensities *I*_i_ and lifetimes τ_i_. Subscript i = 1, 2, and 3 refers to p-Ps, e^+^, and o-Ps, respectively.

#### Potentiometric titrations

Potentiometric titrations were performed in duplicate with a potentiometric system coupled with glass electrode at 25.0 °C. Each titration experiment consisted of approximately 130 successive injections of a concentrated aqueous solution of HNO_3_ (14.0 mol L^–1^) in a beaker loaded with 200 mL of a Fe-Ni/Zn or Fe-Ni/Zn/βCD basic aqueous suspension at an approximate pH of 13.0. The equivalence point was calculated by first-derivative method, which is well established in the literature [46].

#### Zeta potential

ZP measurements were carried out in a Malvern Zetasizer Nano Series ZS (Malvern Instruments, UK) with a 633 nm red laser, through the Malvern Standard M3 technique (with Doppler electrophoresis as the basic principle of operation) by using capillary cell (DPS1060) [37,47]. Average of the ZP values was calculated by ten independent measurements, each one obtained as the mean of 30 counts. ZP values were measured as a function of pH to evaluate the colloidal stability and these measurements were recorded by using different concentrations of nitric acid or sodium hydroxide. ZP titrations were also used to investigate the adsorption process, in which 51 injections of 5.0 μL increments of Cr(NO_3_)_3_ or K_2_Cr_2_O_7_ at 50.0 mmol L^–1^ aqueous solution were titrated into 10.0 mL of Fe-Ni/Zn or Fe-Ni/Zn/βCD at concentrations of 5.1 mg L^–1^ and 4.6 mg L^–1^, respectively. The pH of each solution was not regulated, since the net interaction between the ferrites and the heavy metal could have been disturbed, and moreover, using raw solutions would better represent the conditions in a practical application [4].

#### Sedimentation studies

The kinetic stability of the suspensions was evaluated by relative turbidity determinations as a function of time by using a FEMTO UV–visible spectrophotometer, in the wavelength of 700 nm and with a 1 cm light path quartz cell. Optical obscuration was recorded in intervals of 4 min over 120 min. Suspensions containing 4.6 mg L^–1^ of solid ferrite and 5.1 mg L^–1^ of solid MHM were analyzed at different pH values.

## Supporting Information

File 1 Solid-state characterization of the magnetic hybrid materials.
