# Supplementary material for: Self-assembled organic–inorganic magnetic hybrid adsorbent ferrite based on cyclodextrin nanoparticles
Source: Beilstein J Org Chem. 2012 Nov 1;8:1867–76. doi: 10.3762/bjoc.8.215 (PMC3511024; doi:10.3762/bjoc.8.215)
Supplement: File 1 — Solid-state characterization of the magnetic hybrid materials. [file Beilstein_J_Org_Chem-08-1867-s001.pdf]

Supporting Information

for

Self-assembled organic–inorganic magnetic hybrid adsorbent ferrite based on cyclodextrin nanoparticles

Ângelo M. L. Denadai[1,2], Frederico B. De Sousa[3], Joel J. Passos[3], Fernando C. Guatimosim[3], Kirla D. Barbosa[3], Ana E. Burgos[4], Fernando Castro de Oliveira[1], Jeann C. da Silva[1], Bernardo R. A. Neves[5], Nelcy D. S. Mohallem[6] and Rubén D. Sinisterra[3]*

Address: [1]Centro Federal de Educação Tecnológica (CEFET-MG), Campus VII, Timóteo, MG, Brazil 35183-006, [2]Universidade Federal de Juiz de Fora (UFJF), Governador Valadares, 35010-177, MG, Brazil, [3]Laboratório de Encapsulamento Molecular e Biomateriais (LEMB) – Departamento de Química, Instituto de Ciências Exatas, Universidade Federal de Minas Gerais (UFMG), Belo Horizonte, 31270-901, MG, Brazil, [4]Universidad Nacional de Colombia Bogotá – DC, Colombia, [5]Departamento de Física, ICEx, Universidade Federal de Minas Gerais (UFMG) Belo Horizonte – MG, 31270-901, Brazil and [6]Laboratório de Materiais Nanoestruturados, Departamento de Química, ICEx, Universidade Federal de Minas Gerais (UFMG) Belo Horizonte – MG, 31270-901, Brazil.

Email: Rubén D. Sinisterra* - sinisterra@ufmg.br
*Corresponding author

Solid-state characterization of the magnetic hybrid material

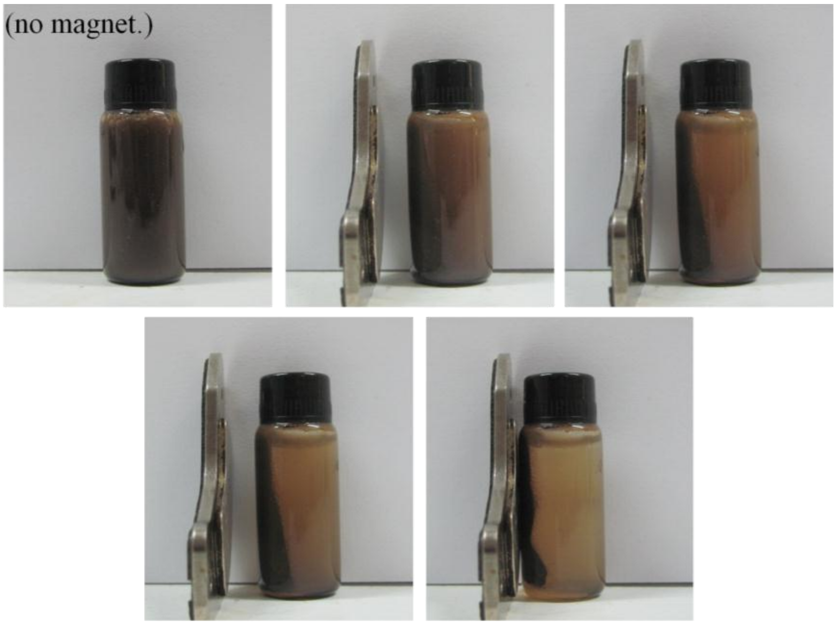

Figure S1: Ferrite magnetic behavior when a magnet is placed close to their suspension as an aqueous solution.

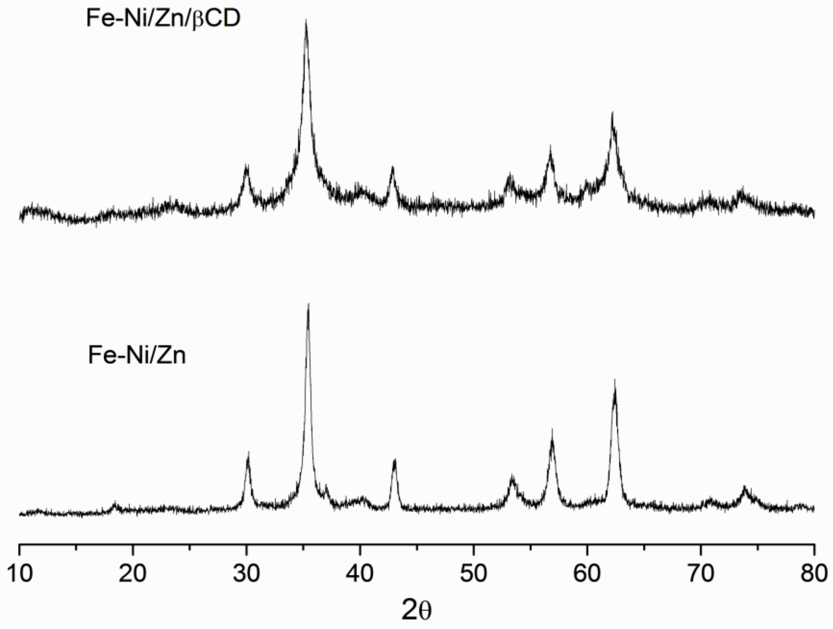

Figure S2: X-ray diffractograms of Fe-Ni/Zn and Fe-Ni/Zn/βCD.

X-ray diffractogram for Fe-Ni/Zn is in accordance with other X-ray powder diffraction analysis described in the literature for Fe-Ni/Zn materials [1,2]. The greater amorphicity observed in the Fe-Ni/Zn/βCD compared to the pure ferrite is due to the βCD incorporated in the inorganic matrix.

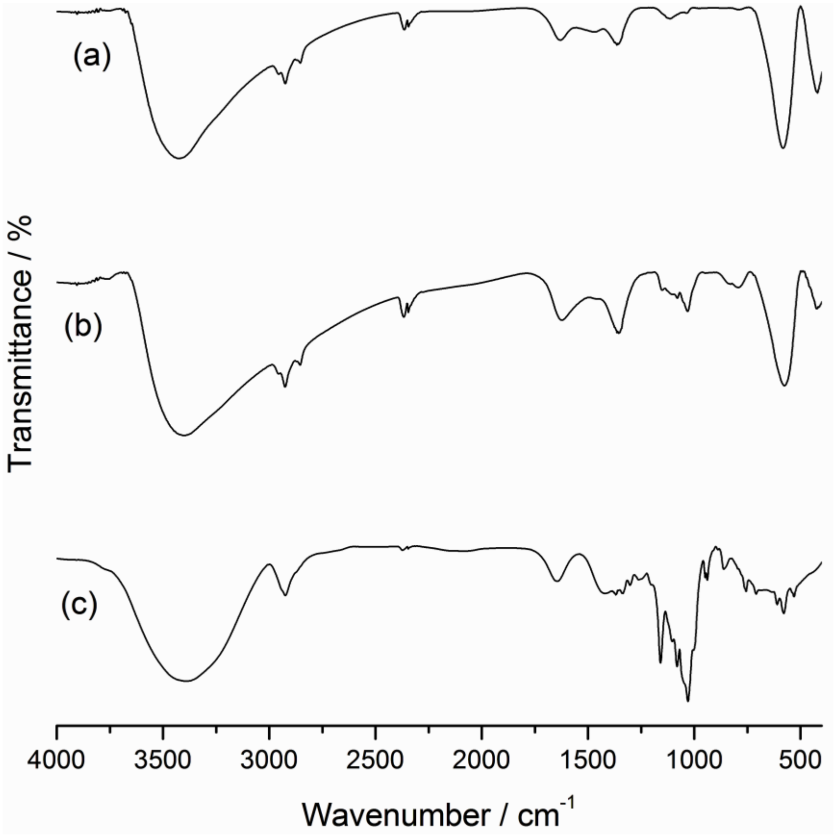

Figure S3: Fourier transformed infrared spectra of: (a) Fe-Ni/Zn, (b) Fe-Ni/Zn/βCD and (c) βCD.

βCD FTIR bands are in accordance with bands previously attributed to this macromolecule [3]. Fe-Ni/Zn/βCD presents the bands associated with the Fe-Ni/Zn and the band at 1050 cm^{-1} from the βCD, which is related to the C–O–C stretching [3]. Fe-Ni/Zn bands are in accordance with previously attribute bands [4,5].

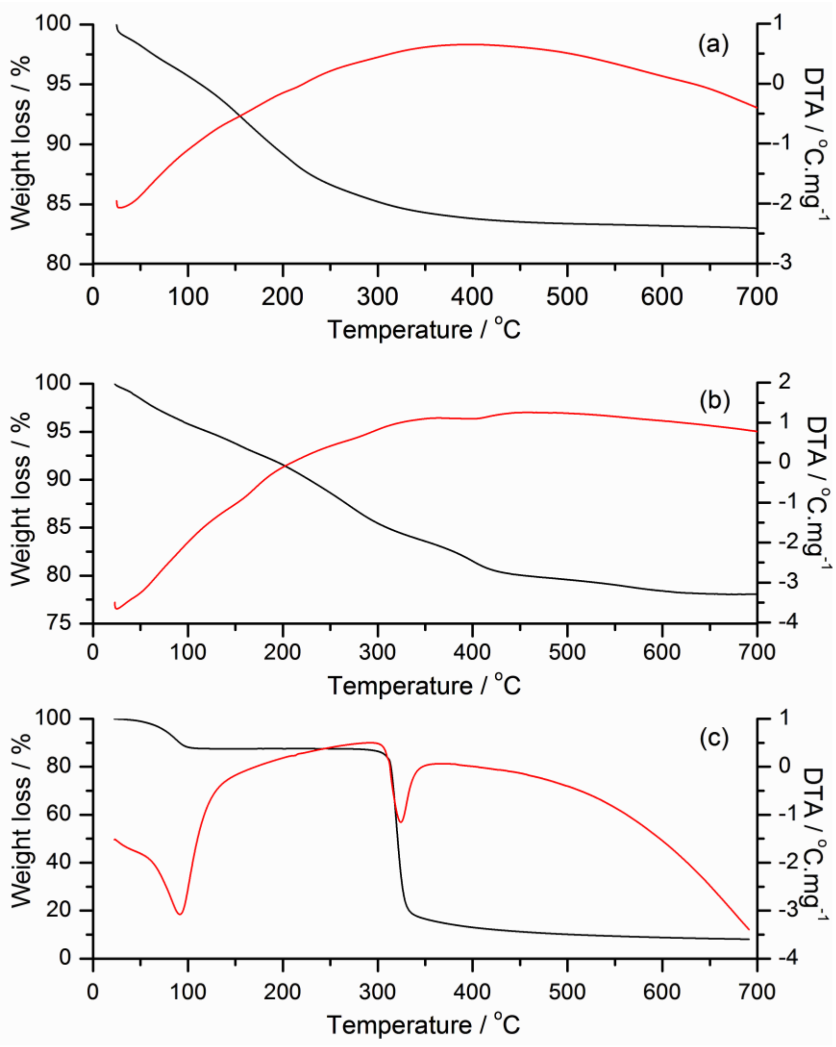

Figure S4: Thermogravimetric analysis (—) and differential thermal analysis (—) of (a) Fe-Ni/Zn, (b) Fe-Ni/Zn/βCD and (c) βCD.

TG and DTA curves of βCD are in agreement with results reported in the literature [6]. The TG and DTA curves of Fe-Ni/Zn are in accordance with work published [7], and the greater weight loss observed in the Fe-Ni/Zn/βCD TG curve could be associated with hydration by water molecules in the MHM matrix due to the βCD incorporation.

This result was confirmed by atomic absorption and elemental analysis (CHN), which allowed us to identify the composition of both ferrites, i.e., $(Fe_2Ni_{0,57}Zn_{0,42}O_4)_{21}(C_{42}H_{70}O_{35})(H_2O)_{28}$ for the MHM and $(Fe_2Ni_{0,54}Zn_{0,51}O_4)(H_2O)_{0,8}$ for the pure ferrite.

References

1. Morrison, S. A.; Cahill, C. L.; Carpenter, E. E.; Calvin, S.; Swaminathan, R.; McHenry, M. E.; Harris, V. G. *J. Appl. Phys.*, **2004**, *95*, 6392.

2. Islam, M. U.; Abbas, T.; Niazi, S. B.; Ahmad, Z.; Sabeen, S.; Chaudhry, M. A. *Solid State Commun.*, **2004**, *130*, 353.

3. Egyed, O. *Vib. Spectrosc.*, **1990**, *1*, 225.

4. Perez, O. P.; Tohji, K.; Umetsu, Y. *J. Alloys Compd.*, **1999**, *290*, 129.

5. Wu, K. H.; Huang, W. C. *J. Solid State Chem.*, **2004**, *177*, 3052.

6. Giordano, F.; Novak, C.; Moyano, J. R. *Thermochim. Acta*, **2001**, *380*, 123.

7. Ramankutty, C. G.; Sugunan, S.; Thomas, B. *J. Mol. Catal. A - Chem.*, **2002**, *187*, 105.
